# Long-term incidence and predictors of in-stent restenosis after chronic total occlusion percutaneous coronary intervention: a cohort study^☆^

**DOI:** 10.1016/j.ijcha.2026.102028

**Published:** 2026-10-14

**Authors:** Lauri Mansikkaniemi, Juhani A. Stewart, Juha Sinisalo, Miia Holmström, Petri Laine

**Affiliations:** a Department of Cardiology, Heart and Lung Center, Helsinki University Hospital and University of Helsinki, Haartmaninkatu 4 (PL 340), Helsinki, 00029, Finland; b Department of Radiology, HUS Diagnostic Center, Helsinki University Hospital and University of Helsinki, Haartmaninkatu 4 (PL 340), Helsinki, 00029, Finland

**Keywords:** Coronary artery disease, Chronic total occlusion, Percutaneous coronary intervention, Drug-eluting stent, In-stent restenosis

## Abstract

**Background:**

In-stent restenosis (ISR) remains a major limitation after percutaneous coronary intervention (PCI) with drug-eluting stents (DESs). However, long-term patency data after chronic total occlusion (CTO) PCI are limited despite high ISR risk. We evaluated long-term ISR incidence and predictors after CTO PCI.

**Methods:**

This prospective single-centre study (NCT07473596) included patients with successful CTO PCI and DES implantation in 2014–2021. Selected survivors underwent protocol-driven surveillance with coronary computed tomography angiography ≥3 years after the index procedure, with suspected ISR confirmed by invasive coronary angiography (ICA). ISR was also identified through clinically indicated ICA during follow-up in all patients. Patient-, lesion-, and procedure-related ISR predictors were analysed.

**Results:**

The study included 294 patients (median age 66 years (IQR 57–74), 79.9% male) and 294 analysed CTO stents. Median clinical and imaging follow-up were 7.1 (IQR 5.1–8.9) and 4.4 (IQR 3.2–6.2) years, respectively. Follow-up imaging covered 186 patients and 186 analysed CTO stents (63.3%). ISR was diagnosed in 40 stents (13.6%). Aalen–Johansen cumulative ISR incidence was 4.8% (95% CI 2.9–7.9), 10.2% (95% CI 7.1–14.5), and 12.9% (95% CI 9.3–18.0) at 3, 5, and 8 years, respectively. Atrial fibrillation (HR 3.71, 95% CI 1.74–7.91, *p* < 0.001) and in-stent CTO (HR 3.51, 95% CI 1.53–8.04, *p* = 0.003) were independently associated with ISR.

**Conclusions:**

Long-term ISR after CTO PCI was infrequent in this high-risk cohort, although estimates should be interpreted in the context of selected imaging follow-up among survivors. ISR was independently associated with atrial fibrillation and in-stent CTO at the index procedure.

## 1 Introduction

Despite major advances in percutaneous coronary intervention (PCI) technology and adjunctive therapy, in-stent restenosis (ISR) remains a clinically relevant limitation after PCI with drug-eluting stents (DESs). ISR accounts for approximately 5–10% of all PCI procedures [1], [2] and has an annual incidence of about 2% [3]. It commonly presents as recurrent angina or acute coronary syndrome and is associated with repeat revascularisation and adverse clinical outcomes [1], [4], [5], [6].

Importantly, ISR often occurs late: nearly half of PCI procedures for ISR are performed more than 2 years after the index intervention [1], and restenotic progression may continue beyond 5 years [7], [8], [9]. Nevertheless, most studies have reported angiographic follow-up of less than 1 year, leaving long-term stent patency insufficiently characterised. This evidence gap is particularly relevant after chronic total occlusion (CTO) PCI, a high-risk setting for ISR [10], [11]. Meta-analyses have reported angiographic ISR rates of 10–11% within the first year after CTO PCI [12], [13]. Beyond the first year, the few available studies have reported ISR rates of 9.9%, 15.6%, and 12.0% at 1.35, 1.50, and 5 years, respectively [14], [15], [16]. Previously reported predictors of ISR include diabetes, chronic kidney disease, small vessel size, longer stent length, a greater number of implanted stents, stent underexpansion, prior myocardial infarction (MI), and prior PCI [4], [17], [18], [19]. In the setting of CTO PCI, a retrograde approach [20], J-CTO score ≥2 [21], and in-stent CTO as the index target lesion [22], [23] have also been associated with ISR.

Contemporary coronary computed tomography angiography (CCTA) offers a useful non-invasive approach for stent follow-up because it can reliably exclude ISR [24], [25], [26], [27]. It is recommended for the follow-up of large-calibre stents in American clinical practice guidelines [28]. This is particularly relevant after CTO PCI, which usually involves major epicardial vessels and large-diameter stents. We have previously demonstrated the feasibility of CCTA for long-term stent patency follow-up after CTO PCI [29].

Accordingly, this study aimed to determine the long-term incidence of angiographic ISR after CTO PCI and to identify factors associated with ISR in this high-risk population.

## 2 Materials and methods

This study was approved by the Ethics Committee of Helsinki University Hospital on 1 June 2022 (approval number HUS/12527/2022). All procedures were performed in accordance with relevant laws, institutional guidelines, and the Declaration of Helsinki. The study was registered retrospectively at ClinicalTrials.gov on 13 March 2026 (NCT07473596), after completion of follow-up but before manuscript submission. The retrospective timing of registration is explicitly acknowledged as a reporting limitation. The manuscript was prepared in accordance with the STROBE Statement [30].

### 2.1 Study population

Study participants were identified from a registry of consecutive patients with stable coronary artery disease and a CTO who underwent PCI at Helsinki University Hospital between 2014 and 2021. Optimal medical therapy was prescribed to all patients at the time of CTO PCI [31], [32]. The study inclusion criterion was a successful CTO PCI with DES implantation at least 3 years before follow-up assessment. If a patient had more than one stented CTO lesion, only the first was included in the analysed cohort.

Stent patency surveillance with CCTA was performed in surviving patients aged ≤80 years at the time of imaging. Patients were consecutive and prospectively recruited, and all patients undergoing surveillance imaging provided written informed consent.

Patients were excluded from protocol-driven surveillance imaging if they had undergone target lesion revascularisation (TLR), had angiographically verified ISR, or had undergone coronary artery bypass surgery after CTO PCI. They were also excluded if ICA had been performed <6 months before the planned surveillance imaging date or if they had an estimated glomerular filtration rate ≤40 mL/min/1.73 m^2^. Further exclusion criteria were malignancy with ongoing diagnostics or poor prognosis; substantial impairment in activities of daily living; memory disorder or other clinically significant cognitive impairment; clinically significant psychiatric disorder; ongoing substance abuse; or inability to communicate in Finnish, Swedish, or English.

Eligibility for protocol-driven surveillance imaging was assessed separately from inclusion in the overall clinical follow-up cohort. Patients who did not undergo protocol-driven surveillance imaging remained eligible for endpoint ascertainment, and data on clinically indicated ICA, ISR, TLR, and other endpoints were retrieved from electronic medical records (EMRs). For patients who died during follow-up, death certificates were obtained from Statistics Finland to determine the cause of death.

In patients with protocol-driven surveillance imaging, CCTA results and clinical data were reviewed by the study interventional cardiologists (JAS and PL). ICA was offered to patients with ISR or an inconclusive finding within the CTO stent. It was also offered to patients with anginal symptoms and either ≥50% stenosis or an inconclusive finding in any native coronary artery or non-CTO stent.

### 2.2 Aims and outcomes

The aim of this study was to describe the long-term (≥3 years) stent patency after CTO PCI and to identify predictors of ISR in this high-risk population. A secondary aim was to identify patients who may benefit from intensified post-procedural follow-up. The primary outcome was the cumulative incidence of long-term ISR after CTO PCI, defined as ≥50% stent luminal narrowing. The secondary outcome was patient-, lesion-, and procedure-related factors associated with ISR. Exploratory clinical endpoints included procedural complications, all-cause death, cardiovascular death, MI, stroke, stent thrombosis, TLR, any coronary revascularisation, and a composite of all-cause death, MI, stroke, and any coronary revascularisation.

### 2.3 Definitions

A coronary CTO was defined as 100% coronary occlusion, with Thrombolysis in Myocardial Infarction (TIMI) flow grade 0 and an estimated duration of ≥3 months. Successful CTO PCI was defined as achievement of TIMI flow grade 3 with residual angiographic stenosis <20%. CTO stents comprised one or more coronary stents, potentially involving different stent types. Stent overlap was taken into account in cases of multiple stents. The diameter of the largest post-dilation balloon or stent was reported. For ISR assessment, the stent segment included the stent and 5 mm proximal and 5 mm distal to the stent, both on CCTA and in ICA [33]. On CCTA, stenosis severity in stents and native coronary arteries was graded in accordance with clinical practice guidelines [34]. ISR was defined as ≥50% luminal narrowing within the CTO stent segment. Clinical endpoints were defined according to the Academic Research Consortium-2 Consensus Document [35].

For CCTA, non-contrast calcium scoring was followed by third-generation dual-source CCTA (Somatom Force, Siemens) using contrast medium (Omnipaque 350, GE Healthcare). Sublingual nitroglycerin was administered before scanning. The imaging protocol was optimised according to heart rate, and when the heart rate exceeded 70/min, intravenous metoprolol was administered.

Patients' symptomatic status at surveillance imaging was assessed by telephone interview (LM) using the Canadian Cardiovascular Society (CCS) classification. For patients without study-driven surveillance imaging, the CCS class at clinically indicated follow-up imaging was determined from EMRs. Patients with CCS class 0 or 1 were considered asymptomatic.

In ICA, ISR was defined as luminal narrowing of ≥50% within the stent segment [36]. Intravascular imaging (intravascular ultrasound (IVUS), optical coherence tomography (OCT)) was performed at the discretion of the interventional cardiologist. Any incidental findings were evaluated separately.

### 2.4 Statistical analysis

Continuous variables were presented as median (IQR), except stent length, which was presented as median (range). Categorical variables were presented as n (%). Kaplan–Meier curves with two-sided 95% pointwise confidence intervals were used to analyse all-cause death and the composite clinical outcome. Cumulative incidence functions were estimated using the Aalen–Johansen method for ISR, non-fatal clinical outcomes, and cardiovascular death in the presence of competing events. These outcomes were presented as cumulative incidence estimates.

For ISR, death before ISR and TLR without prior ISR were treated as competing events. For non-fatal clinical outcomes, death before the outcome of interest was treated as a competing event. For cardiovascular death, non-cardiovascular death was treated as a competing event. For stent thrombosis, death and TLR before stent thrombosis were treated as competing events. Patients without the outcome of interest or a competing event were censored at the end of follow-up.

To avoid clustering from multiple CTO stents within the same patient, one CTO stent was analysed per patient. ISR, TLR, and stent thrombosis were assessed for the analysed CTO stent, whereas mortality and other clinical outcomes were assessed per patient. Predictors of ISR were assessed using cause-specific Cox proportional hazards regression among patients with follow-up imaging available. Univariable Cox models were first fitted for candidate baseline patient-, lesion-, and procedure-related variables. Univariable *p*-values were adjusted using the Benjamini–Hochberg method to account for multiple testing, and variables remaining significant after correction were entered into the multivariable Cox model. Cox regression was not estimated for variables with sparse data or complete separation. Sparse data were defined as fewer than five observations in either predictor category. Complete separation was defined as the absence of either ISR or non-ISR observations in one category. A two-sided *p*-value < 0.05 was considered statistically significant. There were no missing data, and no imputation was performed. Statistical analyses were performed using JMP version 18.2.0 (JMP Statistical Discovery LLC), R version 4.6.1 (R Foundation for Statistical Computing), and RStudio version 2026.06.0 + 242 (Posit Software, PBC).

## 3 Results

### 3.1 Study population

The study included 294 patients and 294 analysed CTO stents, with one CTO stent analysed per patient (Fig. 1). Median clinical and imaging follow-up times were 7.1 (IQR 5.1–8.9) and 4.4 (IQR 3.2–6.2) years, respectively. Follow-up imaging was available for 186 patients, covering 186 analysed CTO stents (63.3%). At the time of follow-up imaging, 95 patients (51.1%) had CCS class II–IV angina. Of the 186 patients with follow-up imaging, 123 underwent protocol-driven surveillance CCTA and 63 underwent clinically indicated ICA. Among patients undergoing surveillance CCTA, findings were confirmed by ICA in 30 patients (24.4%).

**Fig. 1:**
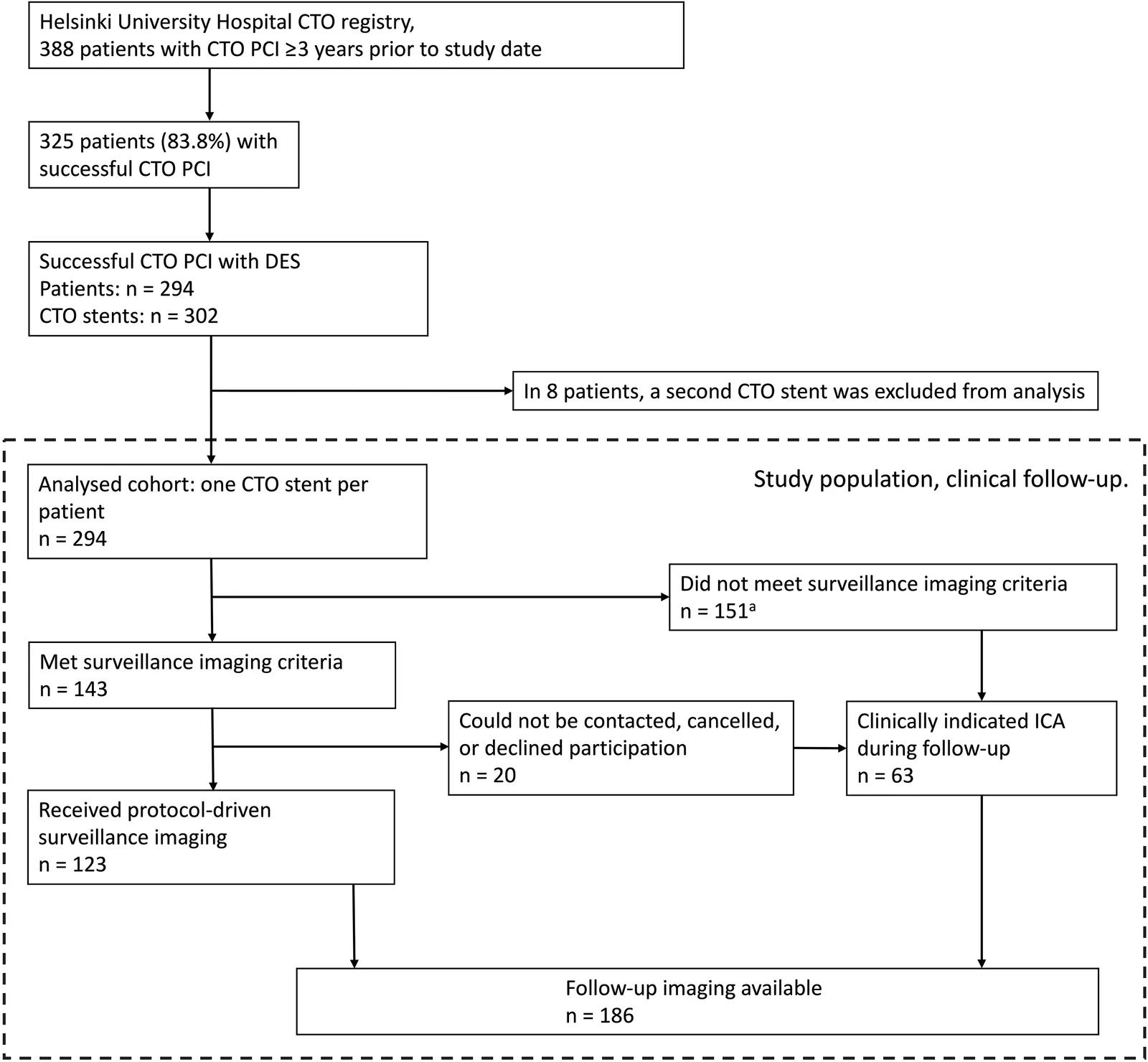
Study flow diagram showing patient selection for clinical follow-up and surveillance imaging. In the analysed cohort, n refers to both patients and analysed CTO stents, as one CTO stent was analysed per patient. ^a^Excluded in hierarchical order due to: death: *n* = 51; age >80 years at surveillance imaging: *n* = 41; TLR or angiographically verified ISR after CTO PCI: *n* = 24; estimated glomerular filtration rate ≤40 mL/min/1.73 m^2^: *n* = 6; memory disorder or other clinically significant cognitive impairment: *n* = 7; substantial impairment in activities of daily living: *n* = 7; ongoing substance abuse: *n* = 4; malignancy with ongoing diagnostics or poor prognosis: *n* = 4; clinically significant psychiatric disorder: *n* = 1; CABG after CTO PCI: *n* = 0; ICA performed <6 months prior to the surveillance imaging date: *n* = 0; inability to communicate in Finnish, Swedish, or English: *n* = 6. CABG: coronary artery bypass surgery; CTO: chronic total occlusion; DES: drug-eluting stent; ICA: invasive coronary angiography; ISR: in-stent restenosis; PCI: percutaneous coronary intervention; TLR: target lesion revascularisation.

Median patient age was 66 (IQR 57–74) years and 79.9% (*n* = 235) patients were male. Baseline patient, lesion, procedural and stent characteristics are shown in Table 1A. All stents were second-generation or newer DES. Xience Pro (Abbott Laboratories) was the most frequently used stent. It accounted for 142 of the CTO stents (48.3%) as the sole stent and contributed to the stent construct in an additional 24 cases (8.2%). Detailed stent types and medications at discharge after CTO PCI are provided in Appendix A, Tables A.1 and A.2.

**Table 1:** Baseline patient, lesion, procedural and stent characteristics at the time of CTO PCI (A) and in-hospital complications (B) and clinical outcomes (C).

A						
Patient characteristics	Patients (*n* = 294)	Lesion characteristics	Lesions (n = 294)	Procedural and stent characteristics	Procedures and stents (n = 294)	
Age, years, median (IQR)	66 (57–74)	Stented vessel		Access		
BMI, kg/m^2^, median (IQR)	27.6 (25.1–31.9)	RCA, n (%)	142 (48.3%)	Radial only, n (%)	98 (33.3%)	
Male, n (%)	235 (79.9%)	LAD, n (%)	104 (35.4%)	Femoral only, n (%)	92 (31.3%)	
Diabetes, n (%)	96 (32.7%)	LCX, n (%)	45 (15.3%)	Radial and femoral, n (%)	104 (35.4%)	
Diabetes on insulin, n (%)	39 (13.3%)	RCA vein graft, n (%)	3 (1.0%)			
Heart failure, n (%)	72 (24.5%)			Antegrade^b^, n (%)	264 (89.8%)	
Peripheral artery disease, n (%)	36 (12.2%)	In-stent CTO, n (%)	13 (4.4%)	Retrograde^c^, n (%)	30 (10.2%)	
Dyslipidemia, n (%)	274 (93.2%)	J-CTO score, median (IQR)	2 (1.0–3.0)	*Re*-entry device^d^, n (%)	4 (1.4%)	
Hypertension, n (%)	239 (81.3%)			Non-CTO lesion PCI, n (%)	31 (10.5%)	
Pulmonary disease, n (%)	50 (17.0%)	J-CTO score^a^, n (%)				
Chronic kidney disease, n (%)	42 (14.3%)	0	25 (8.5%)	Stent		
Atrial fibrillation, n (%)	46 (15.6%)	1	81 (27.6%)	Number of stents, median (IQR)	1.0 (1.0–2.0)	
Cardiac pacemaker, n (%)	11 (3.7%)	2	91 (31.0%)	Diameter, mm, median (IQR)	3.3 (3.0–3.5)	
Smoking history, n (%)	177 (60.2%)	3	44 (15.0%)	Stent length, mm, median (range)	48 (11–160)	
Prior MI, n (%)	112 (38.1%)	4	32 (10.9%)	Single stent, n (%)	170 (57.8%)	
Prior stroke, n (%)	40 (13.6%)	5	21 (7.1%)	Two stents, n (%)	86 (29.3%)	
Prior PCI, n (%)	140 (47.6%)			Three stents, n (%)	30 (10.2%)	
Prior CABG, n (%)	32 (10.9%)			Four stents, n (%)	8 (2.7%)	
Double-CTO, n (%)	9 (3.1%)					
Clinical presentation ACS, n (%)	55 (18.7%)					

**B**						
**In-hospital complications**	Patients (n = 294)					
Access site complication, n (%)	13 (4.4%)					
Coronary perforation, n (%)	1 (0.3%)					
Pericardiocentesis, n (%)	0 (0%)					
LV assist device, n (%)	0 (0%)					
AKI, n (%)	4 (1.4%)					
Stroke, n (%)	1 (0.3%)					
Procedural death, n (%)	0 (0%)					
In-hospital death, n (%)	1 (0.3%)					

**C**						
**Clinical outcomes**	30 days	6 months	1 year	3 years	5 years	8 years
Patient-level outcomes						
All-cause death, n (%)	2 (0.7%)	4 (1.4%)	10 (3.4%)	28 (9.5%)	40 (14.0%)	56 (23.3%)
Cardiovascular death, n (%)	2 (0.7%)	4 (1.4%)	5 (1.7%)	12 (4.1%)	17 (5.9%)	25 (10.3%)
MI, n (%)	2 (0.7%)	5 (1.7%)	9 (3.1%)	16 (5.4%)	24 (8.4%)	32 (13.2%)
Stroke, n (%)	1 (0.3%)	2 (0.7%)	3 (1.0%)	15 (5.1%)	20 (7.1%)	24 (9.1%)
Any coronary revascularisation, n (%)	3 (1.0%)	11 (3.7%)	20 (6.8%)	31 (10.5%)	37 (12.9%)	42 (16.3%)
Combined clinical outcome^e^, n (%)	6 (2.0%)	18 (6.1%)	34 (11.6%)	65 (22.1%)	88 (30.7%)	109 (43.6%)
Stent-level outcomes						
TLR, n (%)	0 (0%)	3 (1.0%)	8 (2.7%)	15 (5.1%)	23 (8.2%)	30 (12.1%)
Stent thrombosis, n (%)	0 (0%)	0 (0%)	1 (0.3%)	4 (1.4%)	4 (1.4%)	4 (1.4%)

Values are presented as median (IQR) or as n (%), as appropriate. Stent length is presented as median and range (minimum–maximum). For panel C, all-cause death and the combined clinical outcome are presented as event count and Kaplan–Meier estimate (%), and all other outcomes as event count and cumulative incidence estimate (%).

ACS: acute coronary syndrome; AKI: acute kidney injury; BMI: body mass index; CABG: coronary artery bypass surgery; CTO: chronic total occlusion; IQR: interquartile range; J-CTO: Japanese chronic total occlusion; LAD: left anterior descending coronary artery; LCX: left circumflex coronary artery; LV: left ventricular; MI: myocardial infarction; PCI: percutaneous coronary intervention; RCA: right coronary artery; TLR: target lesion revascularisation.

a Objective index of procedural difficulty.

b Antegrade wire escalation technique.

c Reverse controlled antegrade and retrograde tracking technique.

d System consisting of the CrossBoss Catheter, Stingray Catheter, and Stingray Guidewire.

e Including all-cause death, MI, stroke, and any coronary revascularisation.

### 3.2 Incidence of ISR

In the analysed cohort, the Aalen–Johansen cumulative incidence of ISR (Fig. 2A) was 4.8% (95% CI 2.9–7.9) at 3 years, 10.2% (95% CI 7.1–14.5) at 5 years, and 12.9% (95% CI 9.3–18.0) at 8 years. ISR was diagnosed in 40 analysed CTO stents (13.6%), with a median time to ISR of 4.0 years (IQR 1.3–5.4). Of these, 15 (37.5%) were first suspected on protocol-driven surveillance CCTA and subsequently confirmed by ICA, whereas 25 (62.5%) were detected by clinically indicated ICA.

**Fig. 2:**
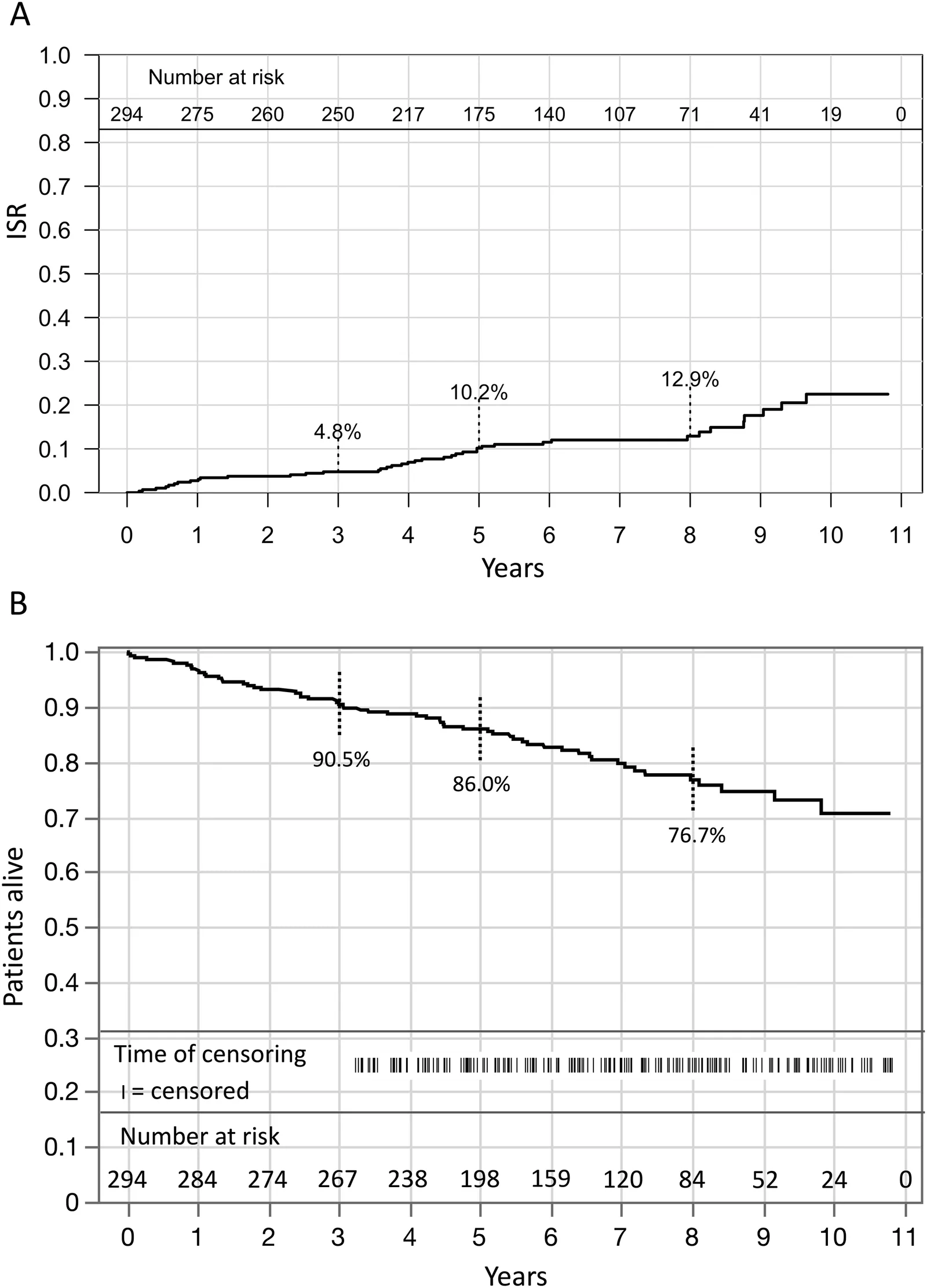
Cumulative incidence function for ISR (A) and Kaplan–Meier curve for all-cause death (B). The Aalen–Johansen cumulative incidence of ISR was 4.8% (95% CI 2.9–7.9) at 3 years, 10.2% (95% CI 7.1–14.5) at 5 years, and 12.9% (95% CI 9.3–18.0) at 8 years. The Kaplan–Meier curve for all-cause death showed a gradual decline in survival over time, with Kaplan–Meier–estimated survival rates of 90.5% (95% CI 86.6–93.3) at 3 years, 86.0% (95% CI 81.5–89.6) at 5 years, and 76.7% (95% CI 70.6–81.9) at 8 years. CI: confidence interval; ISR: in-stent restenosis.

### 3.3 Complications and clinical outcomes

In-hospital complications and clinical outcomes are shown in Table 1, panels B and C, and Fig. 2B. Estimated survival was 90.5% (95% CI 86.6–93.3) at 3 years, 86.0% (95% CI 81.5–89.6) at 5 years, and 76.7% (95% CI 70.6–81.9) at 8 years. During follow-up, 28 patients (9.5%) died from cardiovascular causes. TLR was performed for 34 analysed CTO stents (11.6%). Of the 34 TLR procedures, 9 (26.5%) were performed following ISR detection through protocol-driven surveillance imaging and subsequent clinical assessment. Four TLR procedures (1.4% of analysed CTO stents) were performed for stent thrombosis without prior ISR. In all other TLR cases, ISR was confirmed by ICA before reintervention.

### 3.4 Predictors of in-stent restenosis

Among patients with follow-up imaging available (*n* = 186; ISR events = 40), atrial fibrillation and in-stent CTO remained significant after Benjamini–Hochberg correction and were entered into the multivariable Cox model (Table 2). The final model included two variables, corresponding to 20 events per variable. In multivariable Cox analysis, atrial fibrillation (HR 3.71, 95% CI 1.74–7.91, *p* < 0.001) and in-stent CTO at the index procedure (HR 3.51, 95% CI 1.53–8.04, *p* = 0.003) were independently associated with ISR.

**Table 2:** Cox proportional hazards regression analysis of ISR predictors in 186 stents with follow-up imaging.

	Univariable				Multivariable		
	HR	95% CI	p	p adjusted	HR	95% CI	p
**Patient characteristics**							
Age, years	0.99	0.96–1.03	0.737	0.961			
Sex, female	0.94	0.42–2.14	0.891	0.991			
BMI, kg/m^2^	1.01	0.95–1.08	0.686	0.961			
Prior MI	1.01	0.53–1.90	0.982	0.991			
Prior CABG	2.07	0.87–4.94	0.100	0.390			
Prior stroke	1.97	0.87–4.46	0.104	0.390			
Prior PCI	2.11	1.09–4.08	0.027	0.231			
Atrial fibrillation	4.04	1.90–8.58	<0.001	0.008⁎	3.71	1.74–7.91	<0.001⁎
Dialysis^a^	–	–	–	–			
Smoking history	0.92	0.49–1.71	0.782	0.978			
Peripheral artery disease	1.01	0.31–3.27	0.991	0.991			
Cardiac pacemaker	3.14	1.11–8.85	0.031	0.231			
Prior non-CABG cardiac surgery^b^	–	–	–	–			
Heart failure	0.51	0.20–1.30	0.160	0.436			
Dyslipidemia	0.65	0.20–2.12	0.472	0.885			
Hypertension	1.33	0.56–3.16	0.523	0.923			
Diabetes	1.08	0.55–2.12	0.826	0.991			
Diabetes on insulin	1.27	0.50–3.26	0.616	0.961			
Pulmonary disease	0.83	0.30–2.36	0.732	0.961			
Chronic kidney disease	0.99	0.31–3.22	0.989	0.991			
Clinical presentation ACS	0.85	0.36–2.04	0.720	0.961			

**Lesion and procedural characteristics**							
Target RCA	1.51	0.81–2.83	0.198	0.495			
Target LAD	0.88	0.47–1.65	0.685	0.961			
Target LCX	0.42	0.15–1.17	0.097	0.390			
Target RCA vein graft^c^	–	–	–	–			
In-stent CTO	3.91	1.72–8.92	0.001	0.018⁎	3.51	1.53–8.04	0.003⁎

Number of stents implanted							
Single stent	1.03	0.54–1.98	0.919	0.991			
Two stents	0.73	0.34–1.59	0.427	0.885			
3–4 stents	1.54	0.65–3.68	0.329	0.759			
							
Total stent length, mm	1.00	0.99–1.01	0.465	0.886			
Stent diameter, mm	1.85	0.79–4.32	0.156	0.436			
J-CTO score ≥2	1.96	0.93–4.12	0.075	0.390			
Retrograde approach	1.87	0.78–4.45	0.159	0.436			

The analysis included 40 ISR events. The final multivariable model included two variables, yielding 20 events per variable.

ACS: acute coronary syndrome; BMI: body mass index; CABG: coronary artery bypass surgery; CI: confidence interval; CTO: chronic total occlusion; HR: hazard ratio; ISR: in-stent restenosis; J-CTO: Japanese chronic total occlusion; LAD: left anterior descending coronary artery; LCX: left circumflex coronary artery; MI: myocardial infarction; PCI: percutaneous coronary intervention; RCA: right coronary artery.

⁎ Statistically significant.

a Two patients were undergoing dialysis; neither developed ISR.

b One patient had undergone prior non-CABG cardiac surgery and developed ISR.

c Two analysed CTO stents were located in RCA vein grafts; both developed ISR.

Among patients with follow-up imaging available, ISR was detected in 9 of 21 patients with atrial fibrillation (42.9%) and in 7 of 12 analysed CTO stents treated for in-stent CTO at the index procedure (58.3%).

## 4 Discussion

This prospective, long-term follow-up study yielded two main findings. First, long-term angiographic ISR after CTO PCI was infrequent in this single-centre cohort with mixed clinical and protocol-driven imaging follow-up. Second, atrial fibrillation (HR 3.71) and in-stent CTO at the index procedure (HR 3.51) were independently associated with ISR in the multivariable Cox analysis.

The Aalen–Johansen cumulative incidence of ISR in this high-risk population was 4.8% (95% CI 2.9–7.9) at 3 years, 10.2% (95% CI 7.1–14.5) at 5 years, and 12.9% (95% CI 9.3–18.0) at 8 years (Fig. 2A). In this cohort with selected imaging follow-up, these estimates suggest that the annual ISR burden after CTO PCI with contemporary DES may be modest. Consistent with this favourable long-term profile, rates of adverse clinical events and overall survival were in line with previous literature [37], [38], and in-hospital complication rates were low (Table 1, panels B and C, and Fig. 2B).

Data on angiographic ISR beyond 3 years after CTO PCI remain scarce and are largely limited to studies using first-generation DES, which are associated with worse outcomes than second-generation DES [3]. The PRISON II trial reported a 12.0% ISR rate at 5-year angiographic follow-up after CTO PCI in patients treated with first-generation DES [16], compared with our 5-year estimate of 10.2%. However, the PRISON II analysis included only patients free from major adverse cardiac events, which may have led to underestimation of ISR. A small 3-year angiographic follow-up study reported a higher ISR rate of 13.3%, compared with our estimate of 4.8% [39]. However, the study included only 30 patients. Because longer-term angiographic follow-up data with second-generation or newer DES are limited, studies with shorter follow-up provide relevant context. Nevertheless, comparisons with studies reporting ISR before 3 years require caution, because follow-up before 3 years in the present cohort was clinically driven rather than based on uniform angiographic surveillance. The few available studies including second-generation or newer DES have angiographic follow-up periods shorter than 3 years, reporting ISR rates of 9.9% at 1.35 years [14] and 15.6% at 1.5 years [15]. These rates are not directly comparable with the corresponding estimates in the present cohort (3.4% at 1.35 years and 3.7% at 1.5 years). Asymptomatic ISR before 3 years may have been underdetected, likely contributing to the lower incidence observed at these time points.

Previous studies of non-CTO PCI provide useful context, but direct comparison with the present cohort is limited by differences in study design, patient selection, follow-up intensity, and endpoint ascertainment. The angiographic ISR incidence observed in this study was within the range suggested by previous long-term non-CTO PCI data [40]. It was also consistent with rates inferred from ischaemia-driven TLR after non-CTO PCI, reported at approximately 1–2% per year [3]. Thus, our findings add to the limited long-term evidence on stent patency after CTO PCI and suggest that angiographic restenosis after contemporary CTO PCI may remain infrequent during extended follow-up.

In the final multivariable Cox model, in-stent CTO at the index procedure (HR 3.51, 95% CI 1.53–8.04, *p* = 0.003) and atrial fibrillation (HR 3.71, 95% CI 1.74–7.91, *p* < 0.001) were independently associated with ISR. The association between in-stent CTO and subsequent ISR is consistent with previous studies identifying in-stent CTO as a risk factor for ISR after CTO PCI [22], [23], and with evidence that recurrent ISR becomes more frequent after repeated ISR interventions [41]. Certain patient subgroups, such as those treated for an in-stent CTO, may benefit from targeted counselling at the time of the index procedure regarding the increased risk of ISR. In-stent CTO lesions represent a biologically and mechanically more complex lesion subset. Prior stent failure, multiple stent layers, and greater procedural complexity may contribute to recurrent restenosis. Although in-stent CTO PCI is technically challenging, experienced centres report similar success rates for de novo CTOs and in-stent CTOs [22]. Therefore, recurrent ISR may be driven less by procedural complexity and more by patient- and lesion-related factors underlying restenosis susceptibility. However, the magnitude of the association observed in the present study should be interpreted cautiously, as it was based on only 12 in-stent CTO cases with seven ISR events. Other established risk factors, such as diabetes, were not identified as independent predictors in the present study, possibly because of the already high baseline risk profile of this population.

The association between atrial fibrillation and ISR was unexpected. It should be interpreted cautiously, also taking the wide confidence interval into account. Atrial fibrillation has not been established as a direct causal risk factor for coronary ISR. In the present study, it may instead represent a marker of higher overall comorbidity burden [42] rather than a mechanistic determinant of restenosis. In addition, only 21 of 46 patients with atrial fibrillation in the full analysed cohort had follow-up imaging, which should be considered when interpreting this association.

## 5 Limitations

This study had several limitations. First, the relatively low observed ISR incidence may partly reflect underdetection. Protocol-driven surveillance imaging was performed only after 3 years and was restricted to a selected subset of clinically stable survivors aged ≤80 years with good functional status. Consequently, asymptomatic ISR may have remained undetected or been detected later, and selection of the surveillance cohort may limit the generalisability of the incidence estimates to the broader CTO PCI population. However, patients without protocol-driven surveillance imaging remained in clinical follow-up, and clinically indicated ICA findings and clinical endpoints were included in endpoint ascertainment. Accordingly, findings before 3 years should be interpreted primarily as clinically driven, whereas findings at ≥3 years reflect a combination of clinically and protocol-driven imaging.

Second, negative CCTA findings were not routinely confirmed by ICA, and false-negative findings may therefore have contributed to underestimation of ISR incidence. However, the reliability of a negative CCTA result in stent assessment is high [24], [25], [26], [27], and routine ICA follow-up in asymptomatic patients was not justified for ethical reasons.

Third, imaging follow-up duration varied between patients, potentially reducing the reliability of ISR estimates, although cumulative incidence function analysis was used to account for this. In addition, ISR status could not be confirmed in most of the 28 patients (9.5%) who died from cardiovascular causes, because medical autopsies were infrequently performed. IVUS or OCT was rarely performed at the time of ISR diagnosis, preventing systematic assessment of underlying mechanisms such as stent underexpansion or malapposition.

Finally, the single-centre design may limit generalisability to other centres and operators, while the limited number of ISR events reduced statistical power and increased uncertainty around the effect estimates. The identified associations should therefore be interpreted cautiously. The study was also registered retrospectively after completion of follow-up. Although the surveillance imaging protocol was approved by the institutional ethics committee before recruitment for surveillance imaging, the timing of registration remains a reporting limitation.

## 6 Conclusion

Long-term angiographic ISR after CTO PCI was infrequent in this single-centre cohort, suggesting that the long-term ISR risk may remain low over extended follow-up. However, the incidence estimates should be interpreted in the context of selected imaging follow-up among survivors. Among patients with follow-up imaging, atrial fibrillation and in-stent CTO at the index procedure were independently associated with ISR.

## CRediT authorship contribution statement

**Lauri Mansikkaniemi:** Writing – review & editing, Writing – original draft, Visualization, Methodology, Investigation, Funding acquisition, Formal analysis, Data curation, Conceptualization. **Juhani A. Stewart:** Writing – review & editing, Methodology, Investigation, Conceptualization. **Juha Sinisalo:** Writing – review & editing, Methodology, Conceptualization. **Miia Holmström:** Methodology, Investigation, Funding acquisition, Conceptualization. **Petri Laine:** Writing – review & editing, Methodology, Investigation, Funding acquisition, Conceptualization.

## Funding

This work was supported by the 10.13039/501100005633Finnish Foundation for Cardiovascular Research under grant number 70399 and by a Schmidt Foundation grant under grant number M1055LAHJ1. Open access funded by Helsinki University Library. Author Mansikkaniemi received personal grants from the Paavo Ilmari Ahvenainen Foundation, the Maire Taponen Foundation, the Aarne Koskelo Foundation and the Finnish Society of Angiology. The funders had no role in study design, data collection, analysis, interpretation, manuscript preparation, or the decision to publish.

## Declaration of competing interest

The authors declare that they have no known competing financial interests or personal relationships that could have appeared to influence the work reported in this paper.

## Data Availability

The time-to-event data underlying Fig. 2A and 2B are provided in the supplementary data sheet (Appendix A). Other data underlying this study are available from the corresponding author upon reasonable request and according to the ethical guidelines of Helsinki University Hospital and University of Helsinki.
