# Supplementary material for: Long-term incidence and predictors of in-stent restenosis after chronic total occlusion percutaneous coronary intervention: a cohort study
Source: Int J Cardiol Heart Vasc. 2026 Oct 14;67:102028. doi: 10.1016/j.ijcha.2026.102028 (PMC13694426; doi:10.1016/j.ijcha.2026.102028)
Supplement: Supplementary file 1 [file mmc2.docx]

Table A.1: Stents utilised in the study. All stents were second-generation or newer

| Analysed CTO stents (n = 294) | | | | | | |
| --- | --- | --- | --- | --- | --- | --- |
|  | Only one DES type in the CTO stent (n = 262) | | | | Different DES types combined in the CTO stent (n = 32) | |
|  | 1 stent | 2 stents | 3 stents | 4 stents | Biomatrix Alpha (2 stents), Promus Element | 1 (0.3%) |
| Biomatrix Alpha | 16 (5.4%) | 0 (0%) | 1 (0.3%) | 0 (0%) | Biomatrix Alpha,  Biomatrix Flex | 1 (0.3%) |
| Biomatrix Flex | 1 (0.3%) | 0 (0%) | 0 (0%) | 0 (0%) | Biomatrix Alpha,  Promus Element (2 stents) | 1 (0.3%) |
| Biomatrix Neoflex | 20 (6.8%) | 7 (2.4%) | 0 (0%) | 1 (0.3%) | Biomatrix Alpha,  Promus Premier, Xience Pro | 1 (0.3%) |
| Biomime | 0 (0%) | 1 (0.3%) | 0 (0%) | 0 (0%) | Biomatrix Alpha, Xience Pro | 3 (1.0%) |
| Coroflex Isar Neo | 1 (0.3%) | 0 (0%) | 0 (0%) | 0 (0%) | Biomatrix Flex,  Resolute Onyx | 1 (0.3%) |
| Orsiro | 3 (1.0%) | 0 (0%) | 0 (0%) | 0 (0%) | Biomatrix Neoflex, Biomime, Resolute Onyx | 1 (0.3%) |
| Promus Element | 14 (4.8%) | 0 (0%) | 0 (0%) | 0 (0%) | Biomatrix Neoflex,  Xience Pro | 2 (0.7%) |
| Promus Elite | 2 (0.7%) | 0 (0%) | 0 (0%) | 0 (0%) | Biomatrix Neoflex,  Xience Pro, Xience V | 1 (0.3%) |
| Promus Premier | 13 (4.4%) | 0 (0%) | 0 (0%) | 0 (0%) | Orsiro (2 stents), Xience Pro | 1 (0.3%) |
| Resolute Integrity | 13 (4.4%) | 2 (0.7%) | 2 (0.7%) | 0 (0%) | Promus Element (2 stents), Xience Pro | 1 (0.3%) |
| Resolute Onyx | 4 (1.4%) | 3 (1.0%) | 1 (0.3%) | 1 (0.3%) | Promus Element Plus, Synergy, Xience Pro | 1 (0.3%) |
| Synergy | 2 (0.7%) | 1 (0.3%) | 1 (0.3%) | 0 (0%) | Promus Element, Synergy | 1 (0.3%) |
| Synergy II | 4 (1.4%) | 0 (0%) | 1 (0.3%) | 0 (0%) | Promus Element, Xience Pro | 3 (1.0%) |
| Ultimaster Transei | 2 (0.7%) | 0 (0%) | 0 (0%) | 0 (0%) | Promus Element, Xience Pro (3 stents) | 1 (0.3%) |
| Xience Pro | 96 (32.7%) | 38 (12.9%) | 6 (2.0%) | 2 (0.7%) | Promus Premier (2 stents), Xience Pro | 1 (0.3%) |
| Xience V | 2 (0.7%) | 1 (0.3%) | 0 (0%) | 0 (0%) | Promus Premier, Synergy  (2 stents) | 1 (0.3%) |
|  |  |  |  |  | Promus Premier, Xience Pro | 2 (0.7%) |
|  |  |  |  |  | Promus Premier, Xience Pro (2 stents) | 2 (0.7%) |
|  |  |  |  |  | Resolute Integrity (2 stents), Resolute Onyx (2 stents) | 1 (0.3%) |
|  |  |  |  |  | Resolute Integrity (2 stents), Xience Pro | 1 (0.3%) |
|  |  |  |  |  | Resolute Onyx (2 stents), Xience Pro | 1 (0.3%) |
|  |  |  |  |  | Resolute Onyx, Xience Pro | 1 (0.3%) |
|  |  |  |  |  | Synergy II, Xience Pro  (3 stents) | 1 (0.3%) |
|  |  |  |  |  | Ultimaster Transei, Xience Pro | 1 (0.3%) |
|  |  |  |  |  |  |  |

CTO: chronic total occlusion; DES: drug-eluting stent.

Values are presented as n (%).

Table A.2: Medication at discharge after CTO PCI

|  | Patients (n = 294) |
| --- | --- |
| Aspirin, n (%) | 284 (96.6%) |
| P2Y12 inhibitor, n (%) | 291 (99.0%) |
| Oral anticoagulant, n (%) | 39 (13.3%) |
| Warfarin, n (%) | 14 (4.8%) |
| NOAC, n (%) | 25 (8.5%) |
| Statin, n (%) | 286 (97.3%) |
| Other cholesterol-lowering drug, n (%) | 42 (14.3%) |
| ACEI or ARB, n (%) | 229 (77.9%) |
| Calcium blocker, n (%) | 105 (35.7%) |
| Beta blocker, n (%) | 226 (76.9%) |
| Nitrate, n (%) | 210 (71.4%) |

Values are presented as n (%).

ACEI: angiotensin-converting enzyme inhibitor; ARB: angiotensin II receptor blocker; CTO: chronic total occlusion; NOAC: novel oral anticoagulant; P2Y12: platelet P2Y12 receptor; PCI: percutaneous coronary intervention.
